# Evaluation of the Nordic Musculoskeletal Questionnaire for Measuring Prevalence and the Consequence of Pain in a Danish Adult OI Population: A Pilot Study

**DOI:** 10.1007/s00223-024-01262-9

**Published:** 2024-07-27

**Authors:** Camilla Gehling Horn, Karsten Jensen, Jan Hartvigsen, Lena Lande Wekre, Søren T. Skou, Lars Folkestad

**Affiliations:** 1 Faculty of Health Sciences, Medicine, University of Southern Denmark, Odense, Denmark; 2 The Danish Osteogenesis Imperfecta Society, Tarm, Denmark; 3 Department of Sports Science and Clinical Biomechanics, Center for Muscle and Joint Health, University of Southern Denmark, Odense, Denmark; 4 Chiropractic Knowledge Hub, Odense, Denmark; 5 TRS National Resource Center of Rare Disorders, Sunnaas Rehabilitation Hospital, Bjørnemyr, Norway; 6 The Research and Implementation Unit PROgrez, Department of Physiotherapy and Occupational Therapy, Næstved-Slagelse-Ringsted Hospitals, Slagelse, Denmark; 7 Bone and Mineral Unit, Department of Endocrinology, Odense University Hospital, Odense, Denmark; 8 Department of Clinical Research, University of Southern Denmark, Odense, Denmark

**Keywords:** Osteogenesis Imperfecta, Musculoskeletal pain, The nordic musculoskeletal questionnaire, Analgesics

## Abstract

Pain is a challenge in persons with OI and causes much concern in the Osteogenesis Imperfecta (OI) population. We aim to evaluate the usability of the Nordic Musculoskeletal Questionnaire (NMQ) to identify painful sites in adults with OI and to describe the occurrence of musculoskeletal (MSK) pain and its impact on their work and daily activities. This cross-sectional pilot study uses the OI-NMQ to study MSK pain prevalence in nine separate anatomical regions (neck, upper back, lower back, shoulder, elbow, hand/wrist, hip, knee, and ankle/foot) and its impact on regular work and daily activities in adults with OI. The questionnaire was distributed among participants of the 2023 annual meeting of The Danish OI Society. The response rate was 68%, and all participants considered the OI-NMQ helpful in assessing the presence of pain and its consequences. The analysis included 27 adults with OI type I, III, or IV above 18 years. Among all 27 participants, MSK pain was present in 15–56% of the 9 sites within the last 7 days and 33–89% of the nine anatomical regions during the last 12 months. In 7–48% of all the participants, their regular work and daily activities had been affected by the presence of MSK pain. The OI-NMQ was feasible in assessing MSK pain among adults with OI and displayed a high prevalence of MSK pain with a moderate impact on their regular work and daily activities in this OI population. A larger and repeated measurement of MSK pain in adults with OI is needed to confirm these results.

## Introduction

Osteogenesis Imperfecta (OI) is a heterogenic group of connective tissue disorders with mutations in the genes associated with the biosynthesis of collagen type 1 [1]. OI is a rare disease with a prevalence of 11/100,000 in Denmark and is often grouped according to clinical severity and genetic background using the updated Sillence classification into five phenotypical heterogeneous groups (Type I OI: mild, Type II OI: perinatal lethal, Type III OI: severe, Type IV, and V OI: moderately severe) [2, 3]. All OI types are characterized by recurrent fractures, skeletal deformities, joint hypermobility, hearing impairment, specific comorbidities such as cardiovascular and pulmonary morbidly, and early-onset osteoarthritis [4, 5].

Previous studies in OI have found a high prevalence of musculoskeletal (MSK) pain using tools such as the Visual Analog Scale (VAS), Short Form 36 (SF-36), Short Form 12 (SF-12), Brief Pain Inventory Scale (BPS), and short-form McGill pain Questionnaire 2 (SF-MPQ-2) [6–11]. The back is the most common pain site among people with OI, with a point prevalence of 37–76% [6, 7, 10, 12, 13]. However, few of these tools focus on the consequences of MSK on work and daily activities. The Nordic Musculoskeletal Questionnaire (NMQ) was explicitly designed to evaluate the prevalence and consequences of MSK pain, it has high reliability and validity, and is the most widely used questionnaire to assess MSK pain in the Nordic countries [14, 15]. The NMQ divides the body into nine anatomical regions and consists of three questions regarding MSK pain: the 7-day and annual prevalence of symptoms and the impact on regular work and daily activities [14]. Furthermore, it enables separate assessment of each anatomical region, including specific trends of acute (the past 7 days) and chronic (the past 12 months) MSK pain within a study population.

This pilot study aims to evaluate the NMQ’s usability for studying MSK pain in an OI population and adapt the NMQ to provide information about OI type and treatment. Second, it describes the prevalence of MSK pain and self-reported intake of analgesics as a marker of pain intensity. Finally, we aim to evaluate the consequences of MSK pain on regular work and daily activities among Danish adults with OI by using the NMQ.

## Methods

### Participants

This cross-sectional study included adults diagnosed with OI in Denmark. The eligibility criteria for entering this study were persons above 18 years of age or above with OI who were able to complete the distributed questionnaire.

### Data Collection

Data were collected at The Danish Osteogenesis Imperfecta Society’s annual meeting on May 4th, 2023, after a short introduction of the study aims and the questionnaire. The questionnaire was disseminated in paper form during the meeting and returned to one of the authors (KJ) at end of a 3-day meeting. Participation was voluntary and data were collected anonymously. The questionnaire did not contain any directly identifiable data, such as name, address, or social security number.

### Adaptation of the Questionnaire

An extended OI-NMQ questionnaire was prepared (see appendix) for this study. The first part consisted of anthropometric data of importance for illustrating the participants’ clinical OI type, including gender, age, height, weight, use of aids, number of bone fractures (in the past 12 months), use of fracture-preventive medications, and comorbidities. The OI type was reported using Sillence classification (OI type I–V) based on self-reported clinical phenotypical characteristics and not by their genetics.

The second part comprised the original NMQ [14]. It divides the human body into neck, upper back, lower back, shoulder, elbow, hand/wrist, hip, knee, and foot/ankle and asks whether MSK pain had been present in the past 12 months (yes or no). If the participants answered yes, they were to answer if the MSK pain had been present in the past 7 days (yes or no) and if the MSK pain had prevented them from participating in their regular work and daily activities in the past 12 months (yes or no). MSK pain was described as soreness, pain, discomfort, or numbness. A human body figure outlining the nine anatomical regions was attached to illustrate the symptom sites and avoid misunderstandings regarding what each symptom site comprised.

The third part of the survey consisted of the participants’ use of analgesics (daily, per necessity, never) to highlight if it reflected the prevalence of their MSK pain. The analgesics were selected based on the standard pain medication used for MSK pain and consisted of paracetamol (Anatomical Therapeutic Chemical Classification System Codes: (ATC) N02BE01), non-steroid inflammatory drugs (NSAID, ATC: M01AB05; M01AE01; M01AE02; M01AE51; N02BA01), codeine-containing drugs (ATC: R05DA04; N02AJ06; N02AJ07, N02AJ08; N02AJ09), tramadol (ATC: N02AX02; N02AX06), transdermal and oral opioids (ATC: N02AB03; N02AE01; N02AA01; N02AA03; N02AA05; N02AB01; N02AG02; N07BC02), and other pain medication (ATC: N03AX12; N03AX16; N06AA02; N06AA09; N06AA10; N06AX16; N06AX21) [16, 17].

The final questionnaire was tested for comprehension and face validity first on 4 healthy adult non-OI individuals and then on 4 adults with OI recruited through the Bone and Mineral Unit outpatient clinic at the Department of Endocrinology at Odense University Hospital, to rule out any ambiguity. The test persons were between 30 and 60 years of age and included both males and females. Lastly, the questionnaire was pilot tested at the annual meeting of The Danish Osteogenesis Imperfecta Society, which is the results that will be presented in this paper. The participants were asked to comment on the questionnaire to highlight incomprehensible formulations or possible misunderstandings and suggest improvements.

### Statistical Analyses

Participant characteristics were presented using proportions (*n*) or median [range] as appropriate. The prevalence and consequences of MSK pain were presented as proportions (*n*) with a 95% Confidence Interval (CI) and percentages. We present data from the overall OI population and of two subgroups related to clinical severity.

Type of OI was self-reported using Sillence types, and if missing, the participants were classified using the participants’ height and use of aids as pseudo-markers of severity as indicated by others [2, 18]. We grouped the participants into mild OI if they self-reported having OI type I, or were above 160 cm of height and used no aids. We grouped the participants as having moderate to severe OI if they self-reported having OI type III or IV, or were below 159 cm of height and used any walking aids or wheelchairs. Missing data regarding all questions apart from OI type, height, and weight were considered a negative answer.

Stata (StataCorp LLC, USA) version 18.0 was utilized to manage statistical analyses.

## Results

The questionnaire was distributed to 40 adults with OI, of whom 27 completed questionnaires (response rate 68%) (Table 1). Of these, 14 (9 women) were classified as having mild OI, and 13 (8 women) as having moderate to severe OI. Median age was 47 years [range 36–64]. Participants with moderate to severe OI were shorter and lighter compared to participants with mild OI. A walking stick was the most frequently used aid among persons with mild OI, whereas in moderate to severe OI, the most frequently used aid was a manual or powered wheelchair. Bisphosphonates were more regularly used in moderate to severe OI (*n* = 11, 85%), while calcium and vitamin D were used by 96% of all 27 participants.

**Table 1:** Baseline characteristics of the study population

Characteristics	All *N* = 27	Mild OI^a^ *N* = 14	Moderate to severe OI^b^ N = 13
Women, *n* (%N)	17 (63)	9 (64)	8 (62)
Age [median (Range)], years	47 [36;64]	49 [45;79]	46 [34;55]
Height [median (Range)], cm	152 [143;160]	160 [156;170]	142 [115;146]
Weight [median (Range)], kg	67 [54;80]	73 [64;92]	54 [40;68]
Aids, *n* (%N)			
No aids	8 (30)	7 (50)	1 (8)
Walking stick	5 (19)	3 (21)	2 (15)
Walker	3 (11)	2 (14)	1 (8)
Manual wheelchair	9 (33)	1 (7)	8 (62)
Electric wheelchair	2 (7)	0 (0)	2 (15)
Other aids	7 (26)	3 (21)	4 (31)
Bone fractures^c^, n (%N)			
0	18 (67)	11 (79)	7 (54)
1	4 (15)	1 (7)	3 (23)
2+	2 (7)	1 (7)	1 (8)
Unreported	3 (11)	1 (7)	2 (15)
Bisphosphonates^d^, *n* (%N)			
Current user	16 (59)	5 (36)	11 (85)
Never used	11 (41)	9 (64)	2 (15)
Calcium and vitamin D, *n* (%N)			
Current user	26 (96)	13 (93)	13 (100)
Never used	1 (4)	1 (7)	0 (0)
Comorbidities, *n* (%N)			
Hypertension	12 (44)	6 (43)	6 (46)
Diabetes mellitus	4 (15)	3 (21)	1 (8)
Osteoarthritis	10 (37)	8 (57)	2 (15)
Fibromyalgia	1 (4)	0 (0)	1 (8)
Hypothyroidism	2 (7)	1 (7)	1 (8)

*OI* Osteogenesis Imperfecta, *n* number

^a^Mild OI: OI type I

^b^Moderate to severe OI: OI type III and IV

^c^Bone fractures in the past 12 months

^d^Bisphosphonates include alendronate, pamidronate and zoledronic acid

### The OI-NMQ

None of the responders described any incomprehensible formulations or possible misunderstandings in the OI-NMQ, and none of their comments indicated that the OI-NMQ could not present their pain experiences over the last year. However, one patient with OI suggested adding a question regarding the intensity of their MSK pain because it, according to the responder, could vary from day to day.

### Prevalence of MSK Pain

The prevalence of back pain in the past 7 days was high in both groups, with the lower back (57% vs. 54%) being the most prevalent in mild OI and the neck (29% vs. 46%) and upper back (36% vs. 38%), the most prevalent in moderate to severe OI (Table 2). Data from the past 12 months showed a similar ranking between the two groups in the lower back (93% vs. 85%), the neck (43% vs. 77%), and the upper back (64% vs. 92%).

**Table 2:** Prevalence of MSK pain in the back (neck, upper, and lower back) and the extremities (shoulder, elbow, hand/wrist, hip, knee, and foot/ankle) in the past 7 days and 12 months

	All OI types *N* = 27	Mild OI^a^ *N* = 14	Moderate to severe OI^b^ *N* = 13
7 days, *n* (%, 95% CI)			
Neck	10 (37, 19;58)	4 (29, 8;58)	6 (46, 19;75)
Upper back	10 (37, 19;58)	5 (36, 13;65)	5 (38, 14;68)
Lower back	15 (56, 35;75)	8 (57, 29;82)	7 (54, 25;81)
Shoulder	8 (30, 14;50)	4 (29, 8;58)	4 (31, 9;61)
Elbow	4 (15, 4.2;33.7)	2 (14, 2;43)	2 (15, 2;45)
Hand/wrist	8 (30, 14;50)	2 (14, 2;43)	6 (46, 19;75)
Hip	10 (37, 19;58)	6 (43, 18;71)	4 (31, 9;61)
Knee	5 (19, 6;38)	4 (29, 8;58)	1 (8, 2;36)
Foot/ankle	10 (37, 19;58)	6 (43, 18;71)	4 (31, 9;61)
12 months, *n*(%*N*, 95% CI)			
Neck	16 (59, 39;78)	6 (43, 18;71)	10 (77, 46;95)
Upper back	21 (78, 58;91)	9 (64, 35;87)	12 (92, 64;100)
Lower back	24 (89, 71;98)	13 (93, 66;100)	11 (85, 55;98)
Shoulder	20 (74, 54;89)	9 (64, 35;87)	11 (85, 55;98)
Elbow	9 (33, 17;54)	3 (21, 5;51)	6 (46, 19;75)
Hand/wrist	14 (52, 32;71)	6 (43, 18;71)	8 (62, 32;86)
Hip	20 (74, 54;89)	9 (64, 35;87)	11 (85, 55;98)
Knee	11 (41, 22;61)	8 (57, 29;82)	3 (23, 5;54)
Foot/ankle	16 (59, 39;78)	8 (57, 29;82)	8 (62, 32;86)

*OI* Osteogenesis Imperfecta, *n* number, *CI* confidence interval

^a^Mild OI: OI type I

^b^Moderate to severe OI: OI type III and IV

**Fig. 1:**
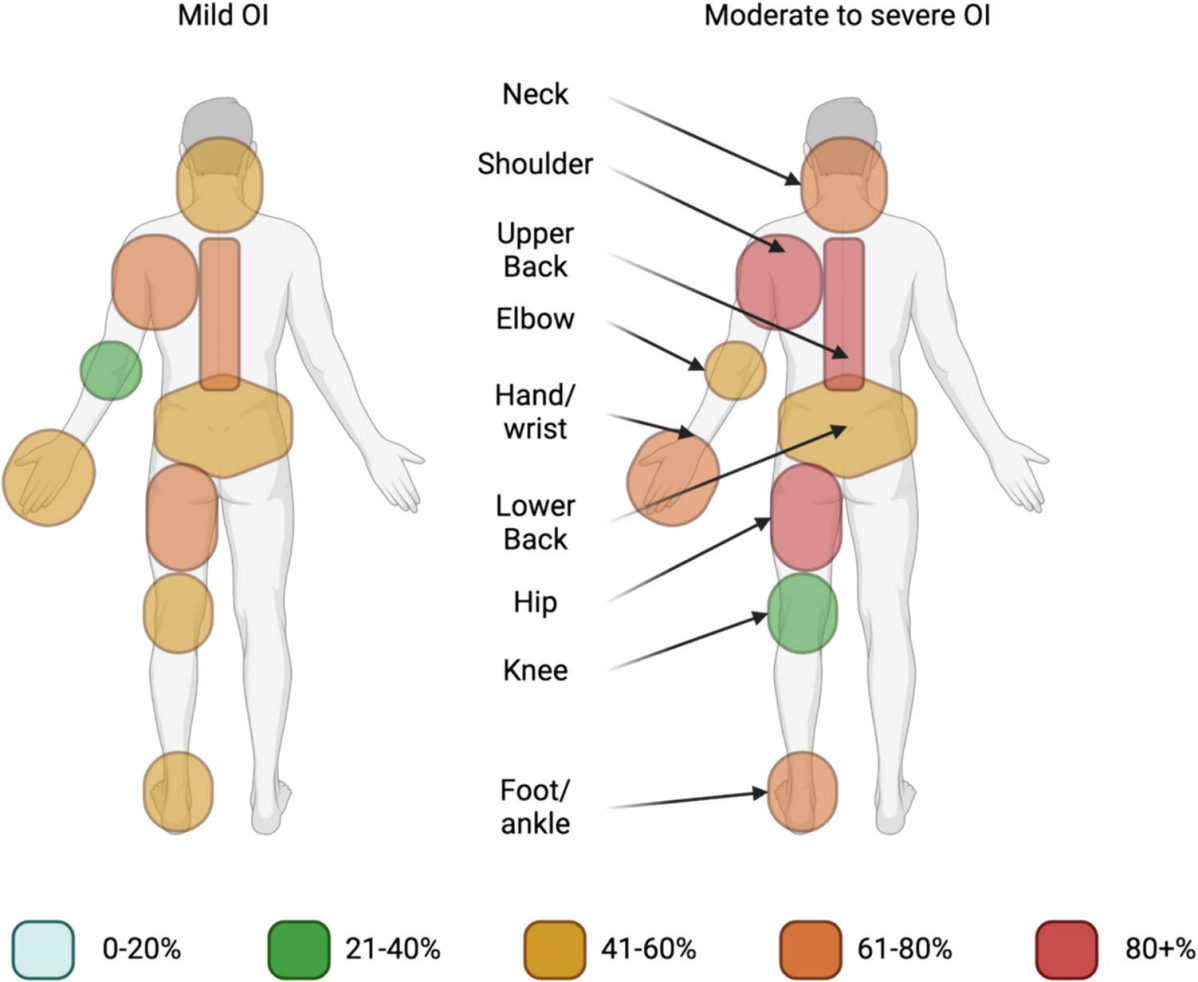
The prevalence of MSK pain, %N, in the past 12 months in OI type I, OI type III, and OI type IV. *n* number, *OI* Osteogenesis Imperfecta, *MSK* musculoskeletal

MSK pain in the extremities varied over the past 7 days between the two groups with higher prevalence in the hip (43% vs. 31%) and foot (43% vs. 31%) in mild OI, and higher prevalence in the hand (14% vs. 46%) among persons with moderate to severe OI. The extremities with the highest prevalence of pain in the past 12 months were the shoulder (64% vs. 85%) and hip (64% vs. 85%) in both anatomical regions. The anatomical regions with the lowest prevalence of pain were the elbow in mild OI (past 7 days 14%, past 12 months 21%) and the knee in moderate to severe OI (past 7 days 8%, past 12 months 23%) (Fig. 1).

The prevalence of MSK pain in the back and extremities was lower during the last 7 days compared to the past 12 months and revealed a variation of MSK pain incidence over time independent of OI severity.

### Use of Analgesics

The use of opioids (daily and per necessity) among persons with mild OI was lower than in persons with moderate to severe OI (7% vs. 46%) (Table 3). Intake of Paracetamol and NSAID’s was similar between the two groups, although more persons with mild OI had a daily intake of Paracetamol than in the persons with moderate to severe OI (42% vs. 23%). Tramadol, transdermal opioids, codeine-containing drugs, and other pain medications were not commonly used in this population regardless of severity group.

**Table 3:** An overview of the study population’s use of analgesics

Pain medication	All OI types *N* = 27	Mild OI^a^ *N* = 14	Moderate to severe OI^b^ *N* = 13
Paracetamol, *n* (%, 95% CI)			
Daily	9 (33, 17;54)	6 (43, 18;71)	3 (23, 5;54)
Per necessity	17 (63, 42;81)	7 (50, 23;77)	10 (77, 46;95)
Never	1 (4, 0;19)	1 (7, 0;34)	0 (0, 0;25)
NSAID, *n* (%, 95% CI)			
Daily	1 (4, 0;19)	1 (7, 0;34)	0 (0, 0;25)
Per necessity	16 (59, 39;78)	7 (50, 23;77)	9 (69, 396;91)
Never	10 (37, 19;58)	6 (43, 18;71)	4 (31, 9;61)
Codeine-containing drugs, *n* (%, 95% CI)			
Daily	0 (0, 0;13)	0 (0, 0;23)	0 (0, 0;25)
Per necessity	2 (7, 9;24)	2 (14, 2;43)	0 (0, 0;25)
Never	25 (93, 76;99)	12 (86, 57;98)	13 (100, 75;100)
Tramadol, *n* (%, 95% CI)			
Daily	2 (7, 9;24)	2 (14, 2;43)	0 (0, 0;25)
Per necessity	3 (11, 2;29)	1 (7, 0;34)	2 (15, 2;45)
Never	22 (81, 62;94)	11 (79, 49;95)	11 (85, 55;98)
Transdermal opioids, *n* (%, 95% CI)			
Daily	1 (4, 0;19)	1 (7, 0;34)	0 (0, 0;25)
Per necessity	0 (0, 0;13)	0 (0, 0;23)	0 (0, 0;25)
Never	26 (96, 81;100)	13 (93, 66;100)	13 (100, 75;100)
Opioids, *n* (%, 95% CI)			
Daily	3 (11, 2;29)	1 (7, 0;34)	2 (15, 2;45)
Per necessity	4 (15, 4;34)	0 (0, 0;23)	4 (31, 9;61)
Never	20 (74, 54;89)	13 (93, 66;100)	7 (54, 25;81)
Other pain medication^c^, *n* (%, 95% CI)			
Daily	3 (11, 2;29)	2 (14, 2;43)	1 (8, 0;36)
Per necessity	0 (0, 0;13)	0 (0, 0;23)	0 (0, 0;25)
Never	24 (89, 71;98)	12 (86, 57;98)	12 (92, 64;100)

*OI* osteogenesis imperfecta *n* number, *CI* confidence interval, *NSAID* non-steroid anti-inflammatory drug, *SNRI* serotonin noradrenalin reuptake inhibitors, *TCA* tricyclic antidepressants

P values are based on a comparison of all three options (daily, per necessity, and never) between the two groups

^a^Mild OI: OI type I

^b^Moderate to severe OI: OI type III and IV

^c^Other pain medication: SNRI, TCA, and anticonvulsants

### The Consequence of MSK Pain

Lower back pain had the highest impact on regular work and daily activities over the past 12 months in both OI patient groups (50% vs. 46%) (Table 4). MSK pain in the neck, elbow, and foot/ankle displayed fewest consequences in persons with mild OI (7%). In participants with moderate to severe OI, the neck and knee presented fewest consequences (8%).

**Table 4:** The prevalence of adults with OI who have been affected in their regular work and daily activities by MSK pain in the past 12 months and to which body region these consequences are related

	All OI types *N* = 27	OI mild^a^ *N* = 14	OI moderate to severe^b^ *N* = 13
Past 12 months, *n* (95% CI)			
Neck	2 (7, 1;24)	1 (7, 0;34)	1 (8, 0;36)
Upper back	6 (22, 9;42)	3 (21, 5;51)	3 (23, 5;54)
Lower back	13 (48, 29;68)	7 (50, 23;77)	6 (46, 19;75)
Shoulder	8 (30, 14;50)	3 (21, 5;51)	5 (38, 14;68)
Elbow	3 (11, 2;29)	1 (7, 0;34)	2 (15, 2;45)
Hand/wrist	4 (15, 4;34)	0 (0, 0;23)	4 (31, 9;61)
Hip	9 (33, 17;54)	3 (21, 5;51)	6 (46, 19;75)
Knee	3 (11, 2;29)	2 (14; 2;43)	1 (8, 0;36)
Foot/ankle	4 (15, 4;34)	1 (7; 0;34)	3 (23, 5;54)

*OI* osteogenesis imperfecta, *n* number, *CI* confidence interval

^a^Mild OI: OI type I

^b^Moderate to severe OI: OI type III and IV

## Discussion

The OI-NMQ was a feasible instrument to assess MSK pain and consequences in persons with OI. The prevalence of MSK pain among persons with OI was generally high in our pilot study, most commonly in the upper and lower back, shoulder, and hip. A larger proportion of participants with moderate to severe OI experienced MSK pain compared to persons with mild OI. The consumption of opioids among persons with moderate to severe OI was higher than in milder OI. MSK pain impacted regular work and daily activities in half of the participants. The OI-NMQ proved to be easily understandable and feasible to use in adults with OI. This assumption is based on the low number of missing answers, few comments related to the questionnaire, and no inconsistencies in the answers related to 7 days and 12 months prevalence of MSK pain. This is in concordance with other studies describing validity and reliability of the NMQ that we have based our questionnaire on [19].

Our study demonstrated a high prevalence of self-reported MSK pain among both groups of adults with OI. MSK pain can be associated with long bone fractures, skeletal deformities, and vertebral fractures in OI [9]. However, only six participants reported one or more fractures during the past year prior to participation and the fracture rate did not correlate to the large proportion of participants experiencing MSK pain during the past 12 months in our study. Thus, indicating that pain can be unrelated to recent fractures in adults with OI. Dove et al. [10] came to the same conclusion that people with OI despite the reporting of new fractures in clinic, the incidence of persistent pain could not be fully explained by current fractures. It is strongly indicating that not all MSK pain in adults with OI is fracture related.

Our questionnaire did not evaluate risk factors, or reasons, for chronic MSK pain in our participants. The high proportion of MSK pain in our study could be caused by the participants previous history of bone fractures and degree of skeletal deformities leading to loss of function and chronic pain, as indicated by others [20–22]. The severity of skeletal deformities is increased in the more severe phenotypes of the disease compared to the milder form [2]. A larger sample size would allow for better comparisons of the prevalence of MSK pain between groups. We must keep in mind that other studies have shown a poor correlation between clinical OI severity and the prevalence of MSK pain [6, 10, 11, 23].

The prevalence of MSK pain in our OI population during the past 7 days of 15–37% in the extremities and 37–56% in the back is higher than for a non-OI population. One study found an overall 14-day pain prevalence of 2.8–12.8% in the extremities (shoulder, elbow, hand/wrist, hip, knee, and foot/ankle) and 6.4–17.6% in the back (neck, lower, and upper back) in a Danish population of 4,817 healthy people aged 16 years of age or above [24]. When evaluating the prevalence of pain during the past 12 months among 20,173 Danish working people, the prevalence of MSK pain was higher, but not as high as in our OI Cohort. Upper-body (neck, shoulder, elbow, hand/wrist) and lower-body (lower back, hip, knee, and foot) MSK pain had a prevalence of 21.4% and 25.5% among working men with no mechanical exposure. For working women with no mechanical exposure, the 14-day MSK pain prevalence was 32.6% for upper-body pain and 28.9% for lower-body pain [25]. A comparative study including a representative and comparable reference population and a large cohort of adults with OI would allow us to evaluate how much higher the prevalence of MSK pain is in the OI population.

Our study found the highest MSK pain prevalence in the past 12 months in the lower back among participants with mild OI (93%) and a similarly high prevalence among participants with moderate to severe OI (85%), which corresponds with other studies of persons with OI [6, 7, 10, 13]. One study of a randomly chosen sample of the Dutch non-OI population found the lower back to be the most common site of MSK pain, with a prevalence of 21.2% [26]. A similar result was found in the Danish study including 20,173 working adults with a lower back MSK pain prevalence of 17.6% [24].

In our study, up to 48% of the participants with OI were missing out on regular work and daily activities due to site-specific MSK pain independent of clinical OI severity. This lack of correlation between clinical severity and the daily consequences of MSK pain and activities of daily living has been shown by others [10, 11]. However, physical Health-Related Quality of Life (HRQoL) has been shown to be significantly lower among adults with type III compared to those with OI type I and IV [11]. In this pilot study, we do not have the statistical power to evaluate between group differences in the consequences of MSK, but the differences in HRQoL observed by other may indicate that there an OI phenotype to MSK pain consequence correlation. Larger studies are needed to evaluate this.

### Limitations and Strength

This study had several limitations. First, the study population of 27 adults with OI is small, and our results may not represent the full Danish or larger international OI population. However, this was a pilot study to evaluate the feasibility of using the OI-NMQ in an OI population, and we have shown consistent results with the current literature by using this tool. Secondly, the OI-NMQ presumed the responder could recall their prevalence of MSK pain for the past 12 months, which might introduce recall bias. Former studies have, however, shown that the answers from the NMQ are reproducible regardless of recall bias [14, 19]. Third, selection bias might affect the results because the inclusion was done at the annual meeting of The Danish Osteogenesis Imperfecta Society. This may result in a study population with a different distribution of severity than what would be expected in the adult Danish OI population. Our study population may, therefore, not reflect the entire adult Danish OI population. However, our results are consistent with the current literature. Lastly, we chose to make missing answers indicative of a negative response, increasing the risk of underestimating the prevalence of MSK pain. Our results should, therefore, be seen as a conservative estimate of the prevalence and consequences of MSK pain in adults with OI. A larger sample would allow for subgroup analysis evaluating differences between clinical sub-types, age groups and genders.

The study is strengthened by the used of the OI-NMQ, a tool developed and adapted to evaluate the prevalence and consequences of MSK pain in an OI population. This is the first study to utilize the NMQ in evaluating MSK pain in adults with OI. Second, the questionnaire was short which made it an easy to understand and quick tool to complete among the participants. The response rate of 68% is acceptable since none of the participants at The Danish Osteogenesis Imperfecta Society annual meeting was pre-informed of the OI-NMQ, and the questionnaire had to be answered and returned by the end of the meeting. At last, the study is strengthened by the close collaboration with The Danish Osteogenesis Imperfecta Society, who have been instrumental in developing the questionnaire, data collection, interpretation, and dissemination.

## Conclusions

We found that the OI-NMQ tool is useful in describing the prevalence and consequences of MSK pain in OI. We found a high prevalence of MSK pain among persons with OI, and higher consumption of opioids was found among persons with moderate to severe OI when compared to those with mild OI. Approximately half of the participants experienced that MSK pain had a negative impact on their regular workability and daily activities during the past year. The causes and consequences of pain are not well understood in people living with OI. Further studies are needed. Using the OI-NMQ tool in a larger cohort, with repeated measurements, would allow us to correlate the prevalence and consequences of MSK to OI phenotype and elaborate on the day-to-day variations of MSK in this population.
